# Seed Germination as an Adaptive Response in Halophytes

**DOI:** 10.3390/plants15111723

**Published:** 2026-06-02

**Authors:** Keriman Şekerci, Nahoko Higashitani, Atsushi Higashitani, Ismail Turkan

**Affiliations:** 1 Graduate School of Life Sciences, Tohoku University, Sendai 980-8577, Japan; nahoko.higashitani.c4@tohoku.ac.jp (N.H.); atsushi.higashitani.e7@tohoku.ac.jp (A.H.); 2 Department of Soil Science and Plant Nutrition, Faculty of Agricultural Sciences and Technologies, Yasar University, Bornova, 35100 Izmir, Türkiye

**Keywords:** halophyte, seed germination, dormancy, recovery, heteromorphism, mucilage

## Abstract

Halophytes thrive in saline habitats through highly specialized adaptive responses, including seed-based strategies to regulate germination timing and ensure reproductive success under fluctuating environmental conditions. Salt-induced quiescence, structural alteration, and regulatory mechanisms are valuable adaptive strategies that facilitate plant growth under high saline conditions, which are becoming increasingly severe due to global climate change. This review provides an overview of the current understanding of halophyte seed germination, dormancy, and recovery. Low to moderate salt exposure generally causes reversible inhibition of germination; however, prolonged or high-level exposure causes stress-induced seed damage. On the other hand, halophyte seeds exhibit regulatory mechanisms associated with germination inhibition under high salt conditions. Adaptive traits such as seed heteromorphism, protective seed coats, mucilage production, and physiological dormancy enhance survival and establishment in saline soils. The ability of halophyte seeds to maintain viability under high salinity and to germinate rapidly when salt stress is alleviated indicates the preservation of metabolic and cellular integrity. Structural adaptations, regulatory mechanisms that balance germination, and the salt-induced quiescent process are controlled by morphological changes and molecular mechanisms. Furthermore, this review highlights the ecological significance and potential applications of halophyte seeds for crop improvement and the restoration of saline and degraded lands. Therefore, understanding the regulatory mechanisms of halophyte seed behavior is a valuable approach for enhancing plant resilience to salinity stress.

## 1. Introduction

Halophytes naturally grow in saline environments and represent approximately 1–2% of the world’s flora; they include both monocot and dicot species (Table 1 and Table 2) [1]. These plants provide valuable genetic information for understanding plant salinity tolerance and improving stress tolerance in crop plants [2,3]. Halophytes can survive in high-salinity environments and exhibit a range of unique morphological, physiological, and reproductive adaptations that enable them to live in hostile environments [4,5,6]. These adaptive behaviors allow halophytes to regulate the timing of germination and seedling establishment so that they occur when the salt concentration decreases, for example during seasonal rainfall. Germination is a process in which coordinated metabolic and molecular mechanisms transform a seed into a developing seedling, ensuring generational continuity and survival.

Halophytes are ecologically and physiologically heterogeneous, and their seed germination strategies are fundamentally shaped by their specific environmental niches. Historically, ecological classifications such as those by Akzhigitova [7] have categorized halophytes based on soil salinity threshold (hyperhalophytes, euhalophytes, hemihalophytes, haloglycophytes) and their response to moisture and groundwater availability (halomesophytes, halohemimesophytes, halomesoxerophytes, and haloxerophytes) [7]. In this review, we bridge these classical ecological groups with modern physiological distinctions. We demonstrate that obligate halophytes such as hyperhalophytes and euhalophytes typically occupy humid saline habitats (halomesophytic niches), where they utilize salt-accumulating strategies for osmotic adjustment. In contrast, facultative halophytes such as haloglycophytes often function as salt excluders, reflecting adaptation to arid environments (haloxerophytic niches), where avoiding ion toxicity and opportunistic germination are key to survival [8].

The functional divergence observed among the halophytes in Table 1 and Table 2 provides a physiological basis for the ecological classifications proposed [7,9]. For instance, the alignment between the taxonomic classification in Table 2 and the functional strategies in Table 1 confirms that obligate species like *Salicornia spp.* are salt accumulators, which serves as a physiological hallmark of hyperhalophytes and euhalophytes adapted to high-moisture, high-salinity environments (halomesophytic niches). In contrast, species categorized as facultative or intermediate halophytes often function as salt secretors and excluders, aligning with the hemihalophyte and haloglycophyte strategies that avoid ionic toxicity in arid habitats. Therefore, seed germination strategies in halophytes should not be interpreted as a single conserved response but rather as adaptive mechanisms shaped by ecological specializations.

Different phylogenetic lineages of halophytes have adapted to germination in distinct ways [10]. As a result, there is no single germination pattern shared by all halophytes. For example, in some monocotyledonous halophytes, germination involves a fruit that is a caryopsis rather than naked seeds. Consequently, germination studies of halophyte seeds should consider not only botanical diversity but also specific structural and functional characteristics, such as seed and fruit tissues, pericarp properties, and tissue-specific adaptations.

**Figure 1:**
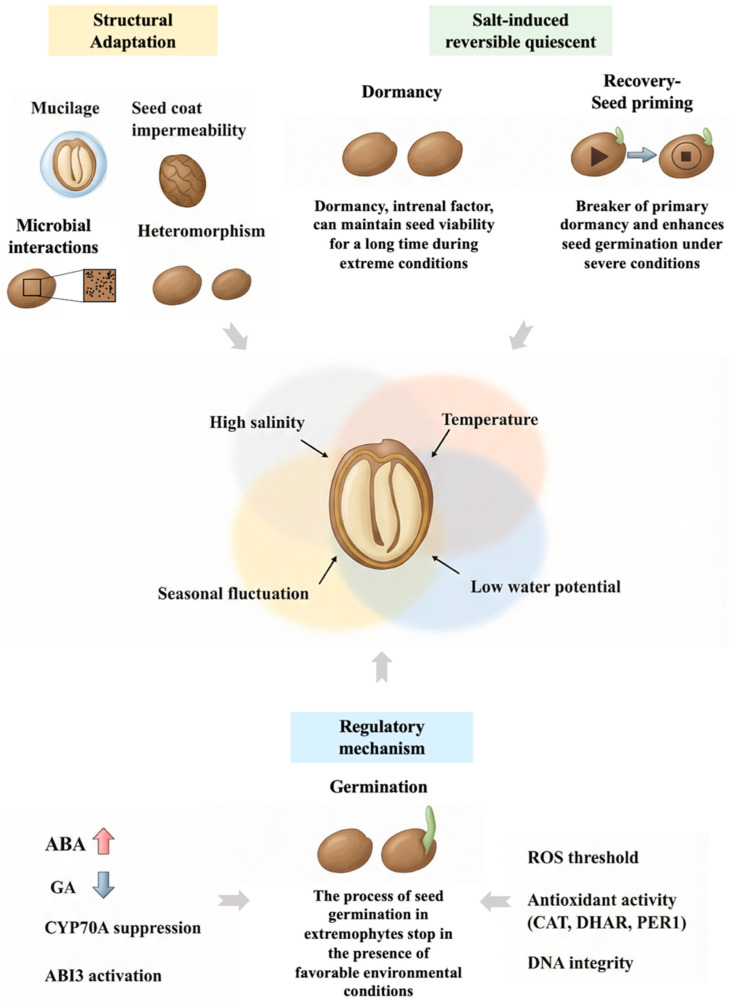
Regulatory mechanisms controlling germination of different halophyte seeds. Halophyte seed germination is not uniform and varies according to species-specific ecological adaptation and seed structural traits. Seed coat permeability, mucilage production, seed heteromorphism, and salt-induced quiescence regulate water uptake, ion exposure, and the transition between inhibition and germination under saline conditions. Halophyte seeds regulate dormancy, recovery, and germination in response to environmental signals, including hormonal factors (ABA/GA), redox status, and structural features. Seed priming mimics this natural recovery process by partially activating pre-germination metabolism and preventing irreversible radicle protrusion.

**Figure 2:**
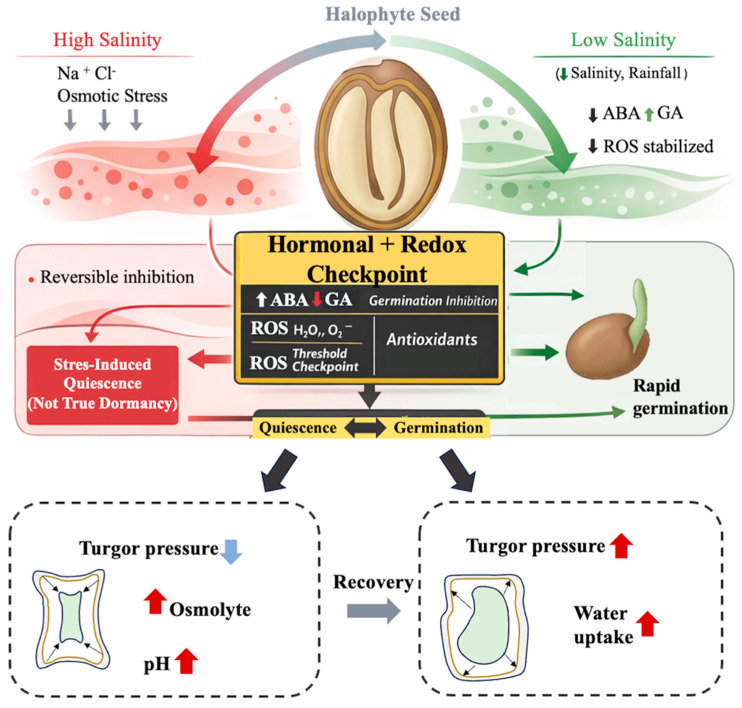
Salinity-induced regulation of different halophyte seed germination. This diagram illustrates the hormonal and redox checkpoints that transition quiescence and germination. Under high salinity, osmotic stress triggers elevated ABA and ROS levels, leading to stress-induced quiescence characterized by low turgor pressure and osmolyte accumulation. When salinity decreases, the balance between ABA and GA is restored, ROS homeostasis is stabilized, and water uptake resumes, allowing rapid germination. This figure highlights the diverse responses of halophyte seeds to saline conditions.

This review suggests that the adaptive behavior of halophyte germination can be illustrated within a three-group adaptation strategy (Figure 1). In the first group, seed structural variables include seed coat permeability, mucilage production, and heteromorphism. These structural factors regulate the entry of water and salt ions into the seed and manage the extent to which the embryo is exposed to salinity during germination. In the second group, physiological regulatory mechanisms, including hormone balance and the coordinated networks involved in osmoregulation and ion homeostasis, are highlighted. The dynamic interaction between the plant hormones abscisic acid (ABA) and gibberellins (GA), together with redox status and stress-responsive transcription factors, determines whether the germination program proceeds or is temporarily suspended under osmotic and ionic stress (Figure 2).

**Table 1:** This table summarizes the adaptive strategies of halophytes. Salt excluders predominantly represent haloxerophytic traits, prioritizing the prevention of ion toxicity in arid habitats, while salt accumulators align with euhalophytic strategies in moisture-rich saline environments.

Species Name	Family	Life Span	Adaptation Strategy	Germination Characteristics
***Aegiceras corniculatum***	Primulaceae	Perennial	Salt secretor	Lost the heme-binding ability of the *DOG1* gene [11]
***Anastatica hierochuntica***	Brassicaceae	Annual	Salt excluder	Hygrochasy; seed dispersal triggered by moisture [12]
***Anthrocnemum macrostachyum***	Chenopodiaceae	Perennial	Salt accumulator	High recovery capacity after hypersaline exposure [13]
***Anthrocnemum meridionale***	Chenopodiaceae	Perennial	Salt accumulator	Nutrient-rich seeds (K, Ca, Mg) for early seedling vigor [14]
***Artemisia sphaerocephala***	Asteraceae	Perennial	Salt excluder	Seed mucilage enhances water retention and soil anchorage [15]
***Cakile maritima***	Brassicaceae	Annual	Salt accumulator	Sea-dispersed seeds; dimorphic fruits for long/short distance dispersal [16]
***Carrichtera annua***	Brassicaceae	Annual	Salt excluder	Opportunistic germination synchronized with rare rainfall [17]
***Chenopodium album***	Amaranthaceae	Annual	Salt excluder	Nitrophilous and salt-tolerant; high plasticity in germination [18]
***Chenopodium quinoa***	Amaranthaceae	Annual	Salt accumulator	Facultative halophyte; seed coat contains saponins for protection [19]
***Colobanthus quitensis***	Brassicaceae	Perennial	Salt excluder	Adapted to low temperatures; metabolic stability [20]
***Diptychocarpus strictus***	Brassicaceae	Annual	Salt excluder	Fruit and seed heteromorphism to spread risk [21]
***Eutrema salsugineum***	Brassicaceae	Perennial	Salt excluder	Coat-imposed dormancy and ABA-mediated control [22]
***Gypsophila oblanceolata***	Caryophyllaceae	Perennial	Salt excluder	Change antioxidant metabolism [23]
***Haloxylon ammodendron***	Amaranthaceae	Perennial	Salt accumulator	Rapid germination to exploit brief moisture [24]
***Haloxylon persicum***	Amaranthaceae	Perennial	Salt excluder	Specialized for sand stabilization; ultra-fast germination [25]
***Kalidium caspicum***	Amaranthaceae	Perennial	Salt accumulator	Highly succulent stems; seeds exhibit deep quiescence under salt [26]
***Lasiurus scindicus***	Poaceae	Perennial	Salt excluder	Synthesis of antimicrobial silver nanoparticles in seeds [27]
***Lepidium perfoliatum***	Brassicaceae	Annual	Salt excluder	Myxospermy; ecological role in sand stabilization [28]
***Limonium bicolor***	Plumbaginaceae	Perennial	Salt secretor	Salt effects seed protein and sugar [29]
***Lobularia maritima***	Brassicaceae	Annual/Perennial	Salt accumulator	Coastal adaptation, rapid recovery [30]
***Panicum turgidum***	Poaceae	Perennial	Salt excluder	Biogenic agent for the nanoparticles [27]
***Plantago spp.***	Plantaginaceae	Perennial	Salt accumulator	Seed mucilage facilitates soil attachment [31]
***Prosopis koelziana***	Fabaceae	Perennial	Salt accumulator	Physical dormancy; plasma-enhanced germination [32]
***Salicornia* spp.**	Amaranthaceae	Annual/Perennial	Salt accumulator	Hypersaline coastal adaptation and high seed oil content [33]
***Salsola ferganica***	Amaranthaceae	Annual	Salt accumulator	Triple seed morphs with hierarchical germination [34]
***Schrenkiella parvula***	Brassicaceae	Annual	Salt accumulator	Reversible salt-induced quiescence via mucilage [35]
***Spergularia salina***	Caryophyllaceae	Annual/Perennial	Salt accumulator	Seed polymorphism (winged vs. wingless) for dispersal strategies [36]
***Suaeda aralocaspica***	Amaranthaceae	Annual	Salt accumulator	Non-centralized embryo; black/brown seed dimorphism [37]
***Suaeda corniculata***	Amaranthaceae	Annual	Salt accumulator	Melatonin-mediated antioxidant defense [38]
***Suaeda glauca***	Amaranthaceae	Annual	Salt accumulator	Dimorphic seeds (black/brown) with varying dormancy levels [39]
***Suaeda liaotungensis***	Amaranthaceae	Annual	Salt accumulator	Pigmentation genes (*ANS, BAN*) regulate seed types [40]
***Suaeda physophora***	Amaranthaceae	Perennial	Salt accumulator	Woody halophyte; perennial strategy to survive chronic salinity [25]
***Reaumuria soongorica***	Tamaricaceae	Perennial	Salt secretor	miRNA-mediated regulation (MIR4995) of ABA catabolism under salt stress [41]

In the third group, salt-induced quiescence represents an environmentally imposed suppression of germination that enables the temporary preservation of seeds under saline conditions. When external salinity decreases, halophyte seeds resume germination, indicating a flexible regulatory mechanism of quiescence rather than a fixed dormancy cycle. A clear distinction between true physiological dormancy and high salinity-induced quiescence must be recognized. Seeds exposed to salinity often exhibit quiescence, in which germination is temporarily inhibited by osmotic and ionic stress rather than by a developmental physiological block. The biophysical state of water within the seeds is primarily responsible for this phenomenon. Water can be found in various forms, including tightly bound water associated with the cell wall and free water. The rate at which the proportion of these water states changes is the most important factor governing water imbibition by the seed and embryo expansion. In addition, most processes involved in water imbibition occur through physical swelling and elongation (expansion) of the seed cells before an energy-consuming stage of cell division occurs.

Based on the information presented above, it is plausible to suggest that halophyte seeds represent a highly effective component of the overall adaptive strategies of plants inhabiting diverse harsh environments, which are rapidly increasing due to human activity [42]. Therefore, understanding the germination mechanisms of halophyte seeds is important for food security, economic sustainability, and the preservation of ecological diversity [43]. Taken together, this knowledge provides an integrated framework for future research in halophyte biology and sustainable agriculture in salt-affected ecosystems [44].

## 2. Factors Involved in Halophyte Seeds Under Saline Environments

### 2.1. Detailed Analysis of Halophyte Seed Coats

The seed coat protects the embryo, prevents desiccation, regulates water uptake, facilitates embryo dispersal, and controls recovery and germination [45,46]. Halophyte seeds have evolved specialized structures that allow them to withstand harsh environments and survive under high-saline conditions [47]. Many halophyte species possess thick seed coats that play a vital role in protecting the embryo and limiting the entry of water, salt ions, and gases. These structural features can contribute to the induction or maintenance of dormancy until environmental conditions become favorable [1,48,49].

Under high-salinity conditions, seeds of the obligate halophyte *Eutrema salsugineum* fail to germinate; however, removal of the seed coat significantly enhances germination capacity [22]. This suggests that the seed coat imposes a mechanical constraint that the embryo cannot overcome under salt stress rather than acting as a barrier to water uptake. Similar structural adaptations have been reported in species of the Amaranthaceae [39,50].

Several halophytes have evolved mechanisms to compartmentalize Na^+^ within their seed coats, along with variations in wax content, both of which are thought to reduce ion toxicity in the embryo under high salinity [25,49]. In addition to serving as a physical barrier, the seed coat of desert halophytes exhibits antimicrobial activity [51].

Molecular analyses of halophyte seed coats are still limited. However, studies in *Arabidopsis thaliana* have shown that the *APETALA2* (*AP2)* gene regulates differentiation of the outer layers of the seed coat, and homologs of this gene have been identified in several halophytes [52,53]. Whether *AP2* homologs play a similar regulatory role in the seed coat of halophytes remains an important area for future research.

Altogether, these findings indicate that the seed coat in halophytes functions not only as a physical barrier but also as a regulator to modulate germination, dormancy-related processes, and stress tolerance under saline conditions.

### 2.2. Seed Mucilage

Seed mucilage is a structural trait of the seed coat, with key functional roles in seed germination. The mucilage produced by halophyte seeds is composed of polysaccharides containing pectin, cellulose, and hemicellulose [54]. This mucilage assists seed survival by promoting hydration, providing physical protection, and aiding seed dispersal [55]. It also helps seeds maintain moisture in arid or semi-arid environments [56], therefore protecting seeds from drought stress [57].

In addition, mucilage influences plant distribution by anchoring seeds to the soil matrix, regulating germination by limiting air diffusion around the seed, and facilitating dispersal through adhesion to animals [28,58,59,60]. Despite these important roles, the literature on mucilage structure and quantity in halophytes under high salinity stress remains limited. In the facultative halophyte *Schrenkiella parvula*, mucilage production is reduced and its architecture is restructured under salt stress, indicating a protective strategy that limits excessive ion uptake [35]. This plasticity may represent a salt-adaptive trait, and mucilage volume and biochemical composition influence hydration dynamics and ion exchange at the seed surface. Mucilage not only facilitates hydration but also provides protection against desiccation. Its adhesive properties enable mucilage-coated seeds to adhere to substrates, which may help camouflage and protect them from herbivores [60,61,62]. The highly branched structures of mucilage, composed of various sugars and acids containing hydroxyl groups, form hydrogen bonds that enhance adhesion. These functional groups promote strong interactions with the substrate surface. Under wet conditions, moisture activates adhesion, allowing mucilage to nonspecifically adhere to a wide range of surfaces [54]. In addition, temperature affects mucilage-binding capacity [60].

Although the ecological roles of mucilage in seed hydration, protection, and dispersal are well recognized, its molecular production in halophytes remains poorly understood. Comparative genomic studies have identified *MYB52* as a positively selected gene involved in seed mucilage production in certain halophytes, such as *E. salsugineum* and *S. parvula* [52]. In *A. thaliana*, the MYB-bHLH-WD40 (MBW) complex and *AP2* regulate primary mucilage biosynthesis [63]. However, whether these regulatory components perform stress-specific functions in halophytes remains unclear. Understanding whether these regulatory modules have been modified or co-opted for salinity adaptation is a critical research direction in halophyte seed biology.

### 2.3. Seed Heteromorphism and Its Effect on Halophyte Seed Adaptation to Saline Environments

Seed heteromorphism, in which a single plant produces two or more distinct seed types differing in color, size, shape, or germination responses, is considered an important bet-hedging strategy that enhances survival in unpredictable and stressful environments [36,64]. Heteromorphic seeds, which exhibit differential germination behavior, enable populations to persist under varying environmental conditions and reduce the risk of complete reproductive failure [2,37,64,65,66]. This characteristic is influenced by environmental factors such as annual precipitation, temperature, photoperiod, and nutrient availability [18,67]. Seed heteromorphism occurs in diverse plant families, including Poaceae, Brassicaceae, and Amaranthaceae [68,69,70,71] (Table 1).

There are several examples that demonstrate its ecological importance. For instance, in the annual obligate halophyte *Salsola ferganica*, three seed morphs exhibit distinct germination percentages that enhance population persistence under variable environmental conditions [72]. The obligate halophyte *Suaeda aralocaspica* produces two seed morphs that differ in shape and embryo pigmentation [37]. Similarly, in the facultative halophyte *Chenopodium quitensis*, variations in seed color have been associated with differences in germination percentage and increased salinity tolerance [66].

Biochemical and molecular parameters further support functional diversity among seed morphs. Differences in soluble sugar and protein content, total nitrogen levels, and antioxidant enzyme activities, including CAT, POD, and proline content, have been associated with distinct salt stress responses [39,40,73]. Genes involved in seed pigmentation, such as *ANS*, *BAN*, and *TT12*, exhibited differential expression between the obligate halophyte *Suaeda liaotungensis* and *S. aralocaspica* seeds when developing in the black- and brown-seed-producing positions on the maternal plant. These findings suggest that these genes are expressed during black seed development [74,75].

Salinity reduces α-amylase activity in glycophytes, thereby decreasing starch degradation and the soluble sugars essential for germination [76]. This reduction in enzyme activity is mainly caused by hormonal changes. Under saline conditions, the gibberellin-to-abscisic acid (GA/ABA) ratio decreases, which leads to the downregulation of α-amylase gene expression [77]. However, halophytes exhibit distinct intraspecific behaviors depending on their seed morphotypes. In heteromorphic species, black seeds often maintain higher α-amylase activity under stress. In contrast, brown seeds with decreased α-amylase activity are more sensitive to salinity [78].

The contrasting physiological responses of black and brown seeds may therefore represent complementary bet-hedging strategies. One morph favors rapid germination under favorable conditions, whereas the other remains more dormant or stress-sensitive, contributing to long-term population stability.

### 2.4. The Role of Seed-Associated Microbiomes Under Salinity

Internal microbial dynamics are crucial to seed development in halophytes. Some studies have shown that the relative abundance of endophytic bacterial taxa differs between the dimorphic seeds of the obligate halophyte *Suaeda glauca* [39]. This divergence is likely driven by differences in seed coat morphology and phytochemical properties. The brown seed coat is soft and membranous, whereas the black seed coat is rigid and has a cuticular exotestal. Consequently, brown seeds favor plant-growth-promoting bacteria, while black seeds host bacteria specializing in enzymatic breakdown, such as pectinase. These distinct microbial communities facilitate the ecological adaptation of dimorphic seeds by performing specialized biological functions.

In addition, arbuscular mycorrhizal fungi (AMF) colonize the emerging radicle and enhance the seedling’s ability to maintain Na^+^/K^+^ balance. Fungal symbionts, such as *Glomus spp.,* reduce oxidative damage in hypersaline germination environments [79]. This symbiotic interaction alleviates osmotic and ionic stress, leading to increases in phosphorus (P) content, biomass, and seed production in the obligate vs. facultative halophyte *Suaeda physophora*. Interestingly, the positive effects of AMF on growth parameters are greater under high salinity than under low-salinity conditions, indicating that the benefits of this symbiosis are enhanced under increasing salinity.

Furthermore, several studies have involved the germination of non-halophyte seeds assisted by halophyte rhizosphere microorganisms known as halotolerant plant growth-promoting rhizobacteria (HT-PGPR), such as *Bacillus*, *Pseudomonas*, and *Halomonas* species, which act as a biological buffer [80]. In particular, microbial production of ACC deaminase reduced Na^+^ concentration in plants and improved water and nutrient absorption. The secretion of IAA promotes rapid radicle emergence and seedling establishment [81]. The direct and indirect effects of PGPR on halophyte seeds should be investigated in future studies, as they may enhance seed germination under salt stress.

## 3. Physiological Balance Mechanisms Regulating Seed Germination in Halophytes Under Salinity

### 3.1. Impact of Saline Environments on Seed Germination

Seed germination represents the initial phase of a plant’s life cycle, facilitating reproduction and ensuring its survival. Germination begins when previously dormant seeds absorb water, a process known as imbibition, and is complete when the radicle emerges from the seed coat. Rapid and synchronized germination is crucial for successful seedling formation and improved crop yield. However, seeds are highly sensitive to environmental fluctuations during germination, and adverse abiotic stress can inhibit seed germination in salt-sensitive plants [82,83].

In contrast, halophytes that can thrive and complete their life cycle in saline environments possess physiological and molecular adaptations [84]. These adaptive responses include ion exclusion by the roots [85], maintenance of ion homeostasis in the leaves [86], and the excretion of toxic ions such as Na^+^ and Cl^−^ through salt glands; thereby, halophytes grow and complete their life cycle under saline conditions [42,87]. However, these adaptive mechanisms are largely inactive during germination. Accordingly, many halophyte species suppress or delay germination under high salinity conditions such as 200–400 mM NaCl, yet their seeds remain viable and readily germinate when the salinity decreases, resulting in an increased substrate water potential [30,88,89,90].

Hormonal regulation, particularly the balance between gibberellins (GA) and abscisic acid (ABA), plays a central role in controlling seed germination under saline conditions [91]. Members of the CYP7070A gene family (*CYP7070A1*, *CYP7070A2*, and *CYP707A3*) encode ABA 8′-hydroxylases that function in ABA catabolism [92,93]. The expression of *CYP7070A1* and *CYP707A3* is significantly downregulated in salt-treated *E. salsugineum* seeds under high NaCl conditions, consistent with increased ABA accumulation [22]. In addition, several studies have demonstrated that miRNA-mediated regulation of ABA biosynthesis genes plays a role in the germination responses of halophytes under NaCl stress [94]. For instance, in seeds of the habitat-indifferent halophyte *Reaumuria soongorica*, *CYP7070A2* has been identified as a potential target of MIR4995 [38]. Moreover, rso-miR499a expression in *R. soongorica* was significantly downregulated at the threshold NaCl concentration of 273 mM compared with the optimal NaCl concentration of 43 mM required for germination, highlighting the regulatory roles of miRNAs and their corresponding mRNAs under salinity [38]. However, salinity-responsive miRNAs and their target regulatory networks in different halophyte cell types remain largely unexplored. Further investigation of these regulatory pathways may contribute to improving plant stress tolerance in economically important crops, which is critical for ensuring future food security under climate change [95].

Comparative transcriptomic analysis between *S. parvula* and *A. thaliana* revealed that genes such as *abscisic acid insensitive 5* (*ABI5*), *RGA-like 2* (*RGL2*), *delay of germination 1* (*DOG1*), and *enolase 2* (*ENO2*), as well as antioxidant-related genes such as *CAT1* and *DHAR2*, were more strongly induced in *S. parvula* seeds than in *A. thaliana* seeds under NaCl treatments [96]. In addition, seed germination regulators such as *PSRP2* in *E. salsugineum* (an At3g52150 ortholog) and *PER1* in *S. parvula* (an At1g48130 ortholog) have been associated with enhanced seed dormancy and have been shown to undergo positive selection [52]. These findings indicate that germination control in halophytes has been evolutionarily optimized.

Although only a limited number of studies have examined reactive oxygen species (ROS) production and responses in halophyte seeds, it has been proposed that the ROS threshold may function as a checkpoint for genome integrity and stress sensing under salinity. Direct measurements of DNA damage during halophyte seed germination have not yet been done; however, several studies indicate that delayed germination or germination recovery under saline conditions is associated with enhanced enzymatic and non-enzymatic antioxidant capacity. These observations support the hypothesis that halophytes suppress germination until ROS levels decline below harmful thresholds, thereby preserving genomic integrity during early development [23].

In addition, under salinity, when the external osmotic potential is very low, special mechanisms enable the seed to absorb water and generate the turgor pressure required for cell wall expansion. For turgor pressure to develop, the seed’s internal osmotic potential must be lower than that of the surrounding saline environment. Halophytes mitigate this through osmotic adjustment, which helps retain intracellular water [97]. If environmental salinity exceeds the threshold below which the seed can maintain turgor, the seed enters a quiescent phase. ABA signaling, which maintains quiescence, is also correlated with turgor modulation [98] (Figure 2). One example is obligate halophyte *Halostachys capsica*, whose seeds are more strongly inhibited by alkaline salts (Na_2_CO_3_) than neutral salts such as NaCl. High pH can damage the cell membrane and disrupt osmotic pressure. If the cell membrane is compromised by high pH, the cell cannot maintain a solute gradient, leading to a permanent loss of turgor. Interestingly, when salt-alkali stress is alleviated by water, the osmotic gradient is rapidly restored, allowing the embryo to break through the seed coat [99]. This structural resilience is linked to halophyte lipid metabolism.

**Figure 3:**
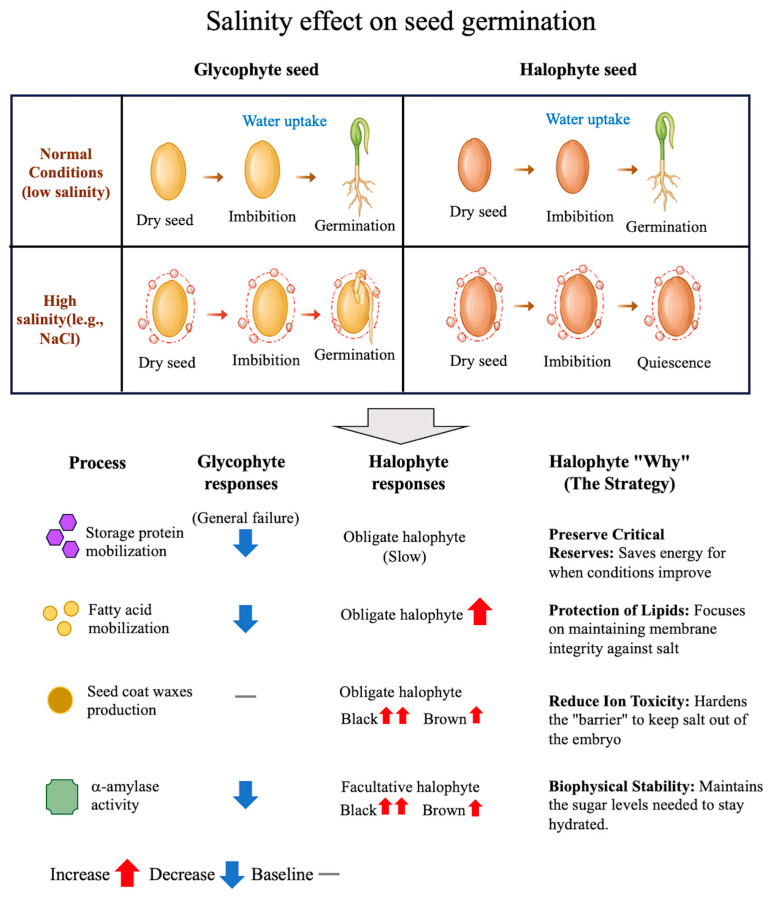
Salinity effects on seed germination in glycophytes and halophytes. This figure compares the contrasting general germination responses of glycophyte seeds and halophyte seeds, including obligate and facultative types, under salt stress. While glycophyte seeds show reduced storage protein mobilization, fatty acid mobilization, and α-amylase activity under high salt conditions, halophyte seeds exhibit adaptive regulation of reserve mobilization, quiescence, and recovery, enabling survival and germination under fluctuating conditions. The red and blue arrows and gray horizontal line indicate an increase, a decrease, and baseline processes, respectively.

Glycophyte seed oil content and membrane integrity are reduced under salt stress [100,101]. However, certain halophyte species such as *Salicornia spp.*, exhibit enhanced oil accumulation under saline conditions (Figure 3) [102]. Halophyte seeds are characterized by a high proportion of essential fatty acids, including alpha-linolenic, linoleic, and palmitic acids, which contribute to membrane stability [103]. Salinity has been demonstrated to enhance the fatty acid content in the dimorphic seeds of the obligate halophyte *Sueda salsa*, while similar conditions reduce fatty acid content in glycophyte seeds (Figure 3) [104].

Furthermore, storage proteins constitute the primary internal nutrient reserves that sustain early seedling establishment. Their mobilization depends on tightly regulated proteolytic activity, which converts stored proteins into free amino acids required for respiration, biosynthesis, and growth [105]. Under saline conditions, this proteolytic machinery is frequently suppressed. Although detailed studies on storage protein dynamics under salinity remain limited, some halophytic species, such as obligate halophyte *Cakile maritima*, exhibit a slower rate of storage protein degradation after salt exposure [106]. This delayed mobilization represents an adaptive strategy to preserve critical reserves during unfavorable conditions (Figure 3).

Together, these processes ensure cellular structures and genetic integrity, enabling seeds to rapidly germinate once favorable conditions are restored (Figure 2).

### 3.2. Seed Germination Responses of Halophytes to Multiple Abiotic Stresses

In natural saline habitats, seeds are rarely exposed to a single stress factor [90]. The effects of multiple stresses can either exacerbate or mitigate their impact on germination. For instance, osmotic and ionic stresses can severely restrict water uptake and delay germination [107]. Halophyte seeds have evolved adaptation strategies that cope with complex environments, including osmotic adaptation, ion homeostasis, and hormonal regulation.

Consistent with these patterns, the response of facultative halophyte sea barley (*Hordeum marinum*) seeds to salinity, temperature, osmotic stress, pH, and waterlogging highlights the regulatory role of abiotic interactions. In particular, optimal temperatures (25 °C) can alleviate salt inhibition, whereas suboptimal temperatures exacerbate it [108]. This environmental sensitivity is modulated by seed dimorphism in *S. salsa*. The brown and black seeds exhibit distinct survival strategies under fluctuating environment conditions.

Brown seeds display broad ecological tolerance, maintaining high germination percentages across a wide temperature range under saline conditions. In contrast, black seeds show a more temperature-dependent response and employ a recovery strategy. When transferred from salt to deionized water, they achieved higher germination rates. This suggests a trade-off between immediate germination and long-term survival, with black seeds delaying germination for more favorable conditions in order to reduce the risk of salt-induced mortality [109].

### 3.3. Integrated Core Mechanisms Regulating Halophyte Seed Germination Under Salinity

(1) Physiological checkpoints: hormonal and redox signals

High salinity elevates ABA levels and suppresses GA activity, thereby enhancing inhibitory signaling. The management of reactive oxygen species (ROS), such as superoxide radicals (O_2_^−^), hydrogen peroxide (H_2_O_2_), and hydroxyl radicals (OH), enhances antioxidants, prevents oxidative damage, and maintains halophyte seed viability. Germination is modulated by the dynamic interaction between hormonal and redox signals, mediated in part by Ca^2+^ signals, nitric oxide (NO), and the MAPK signaling cascade [110]. These hormonal-redox interactions function as physiological checkpoints that determine whether radicle protrusion is delayed or enters a reversible inhibition state.

(2) Molecular adaptation and germination regulators

Comparative genomic analyses of halophytes have identified several genes related to hormone metabolism, ion transport, dormancy regulation, and cell wall remodeling, many of which show signs of positive selection. These genetic adaptations facilitate rapid recovery after stress, allowing seeds to transition from non-germinated to germinated when salinity levels decrease.

In summary, germination reliability under fluctuating salinity is controlled by coordinated physiological and molecular processes that form a regulatory network. This system helps to understand halophyte seed resilience at both ecological and evolutionary levels.

## 4. Salt-Induced Quiescence

### 4.1. Dormancy: A Controlled Survival Strategy in Halophyte Seeds

Seed dormancy occurs during seed development as an evolutionary adaptation that regulates the timing of germination across different stages of seed dispersal. Primary dormancy is established during seed maturation and is typically strongest at the time of dispersal; however, it can be released by specific environmental cues. In contrast, secondary dormancy is induced when previously non-dormant seeds experience environmental conditions that are unfavorable for germination after dispersal [111].

The germination of halophyte seeds may be limited in saline environments; that is, high salinity does not favor seed germination. The osmotic and ionic stress imposed by salinity inhibits water uptake and disrupts seed metabolism [112]. Therefore, salt-induced inhibition should not be confused with “true” physiological dormancy. Although halophyte seeds germinate readily in non-saline water, their germination remains inhibited under saline conditions [30,88,89,90].

Prolonged exposure to high salinity can have both protective and detrimental effects on halophyte seeds. These effects depend on intensity, duration, and environmental context. The failure of these seeds to germinate under saline conditions results from reversible, environmentally induced inhibition. When external salinity decreases, these seeds rapidly resume germination without the need for classical dormancy-breaking treatments [22,35]. Given this response, it is more accurate to describe this behavior as stress-induced quiescence or regulatory arrest rather than dormancy. Several molecular components associated with dormancy regulation, such as ABA signaling pathways and ABI3 expression, may be activated as part of the salt stress response [37,113]. However, the activation of dormancy-related pathways under saline conditions signifies the recruitment of conserved regulatory modules rather than the establishment of a true dormancy state. Thus, although salt stress induces partial convergence with classical dormancy pathways at the level of signal transduction, it does not alter the developmental stage of seed.

True developmental dormancy occurs in some halophytes and may vary among seed morphs within certain taxa. For example, in heteromorphic species, differences in dormancy depth between seed morphs can be associated with structural traits, such as seed coat complexity, hormonal sensitivity, and ecological timing strategies [39,114]. However, these developmental dormancy mechanisms must be distinguished from the germination recovery arrest induced by salinity. Halophytes exhibit a plastic strategy of stress-induced quiescence that temporarily suppresses germination. Distinguishing between physiologically dormant seeds and seeds exhibiting stress-induced inactivity caused by high osmotic potential or ion toxicity can be difficult. Several halophytes remain in a quiescent state under excessive salinity (Table 2) [115,116,117]. Conversely, germination rapidly resumes when salt levels decline below a critical threshold, indicating recovery [118,119]. Therefore, quiescence is a key adaptive strategy that regulates the timing of seed germination.

During the dry, or desiccation, phase, metabolism is reduced to a level sufficient to maintain the seed for extended periods [120]. Hormone precursors, key enzymes, and other regulatory components are primarily produced while the seeds develop on the mother plant. From the moment water enters, it initiates limited metabolic activity, even under saline conditions [121]. In the dry phase, stored reserves are mobilized, and the ABA/GA balance is established. This balance determines whether the seed remains non-germinated or whether the radicle emerges.

Furthermore, the structure of the dispersal unit influences seed dormancy and germination. In many monocotyledonous halophytes, the pericarp (fruit wall) or persistent glumes significantly affect dormancy. These structures act as physical barriers to the environment. Dormancy in these species typically arises from a combination of mechanical constraints on the embryo and the physiological state of the embryo. The metabolic activity involved in the initial phase of germination is already present in the form of hormones and enzymes. These are synthesized and stored during seed development and maturation prior to seed desiccation. Upon rehydration, or imbibition, these stored components are rapidly activated or mobilized rather than being synthesized de novo after drying.

**Table 2:** Classification of species into Obligate and Facultative groups. This physiological distinction reflects the ecological spectrum from hyperhalophytes/euhalophytes (obligate) to haloglycophytes (facultative), illustrating the divergent evolution of germination under varying water tables.

Species Name	Family	Monocot/Dicot	Classification	Salt Concentration
***Acacia tortilis***	Fabaceae	Dicot	Facultative halophyte	0–800 mM NaCl [88]
***Aegiceras corniculatum***	Primulaceae	Dicot	Facultative halophyte	0–400 mM NaCl [87]
***Aeluropus lagopoides***	Poaceae	Monocot	Facultative halophyte	0–800 mM NaCl [88]
***Arthrocnemum indicum***	Amaranthaceae	Dicot	Obligate halophyte	0–1000 mM NaCl [2]
***Arthrocnemum macrostachyum***	Amaranthaceae	Dicot	Obligate halophyte	0–1000 mM NaCl [2]
***Arthrocnemum meridionale***	Amaranthaceae	Dicot	Obligate halophyte	0–200 mM NaCl [14]
***Caroxylon imbricatum***	Amaranthaceae	Dicot	Habitat-independent/ facultative	0–800 mM NaCl [88]
***Chenopodium album***	Amaranthaceae	Dicot	Facultative halophyte	0–400 mM NaCl [18]
***Chenopodium quinoa***	Amaranthaceae	Dicot	Facultative halophyte	0–400 mM NaCl 0–10.2 mM CaCl_2_ 0–10.2 mM KCl 0–53.5 mM MgCl_2_ [121]
***Colobanthus quitensis***	Caryophyllaceae	Dicot	Facultative halophyte	0–200 mM NaCl [66]
***Eutrema salsugineum***	Brassicaceae	Dicot	Obligate halophyte	0–200 mM NaCl 10 mM LiCl [22]
***Glycyrrhiza uralensis***	Fabaceae	Dicot	Facultative halophyte	0–800 mM NaCl [88]
***Gypsophila oblanceolata***	Caryophyllaceae	Dicot	Facultative halophyte	0–300 mM NaCl [23]
***Halogeton glomeratus***	Amaranthaceae	Dicot	Obligate/Facultative	0–800 mM NaCl [88]
***Halopeplis perfoliata***	Chenopodiaceae	Dicot	Obligate halophyte	0–800 mM NaCl [88]
***Haloxylon ammodendron***	Amaranthaceae	Dicot	Facultative halophyte	1.3 M NaCl [122]
***Haloxylon persicum***	Amaranthaceae	Dicot	Facultative halophyte	1.3 M NaCl [122]
***Hordeum marinum***	Poaceae	Monocot	Facultative halophyte	40 mM NaCl [108]
***Kochia scoparia***	Chenopodiaceae	Dicot	Facultative halophyte	1 M NaCl [122]
***Limonium axillare***	Plumbaginaceae	Dicot	Obligate halophyte	0–800 mM NaCl [88]
***Limonium bicolor***	Plumbaginaceae	Dicot	Facultative halophyte	NaCl, Na_2_SO_4_ [29]
***Limonium vulgare***	Plumbaginaceae	Dicot	Facultative halophyte	1.5 M NaCl [122]
***Lobularia maritima***	Brassicaceae	Dicot	Facultative halophyte	50–300 mM NaCl [30]
***Panicum antidotale***	Poaceae	Monocot	Facultative halophyte	0–150 mM NaCl [123]
***Portulaca oleracea***	Portulacaceae	Dicot	Facultative halophyte	0–400 mM NaCl [123]
***Prosopis koelziana***	Fabaceae	Dicot	Facultative halophyte	100–200 mM NaCl [32]
***Reaumuria soongorica***	Tamaricaceae	Dicot	Facultative halophyte	43 mM NaCl [41]
***Suaeda fruticosa***	Amaranthaceae	Dicot	Obligate halophyte	0–400 mM NaCl [123]
***Salicornia rubra***	Amaranthaceae	Dicot	Obligate halophyte	1 M NaCl [122]
***Salsola ferganica***	Amaranthaceae	Dicot	Obligate halophyte	0–1000 mM NaCl [34]
***Salsola iberica***	Amaranthaceae	Dicot	Facultative halophyte	1 M NaCl [122]
***Salsola setifera***	Amaranthaceae	Dicot	Facultative halophyte	0–800 mM NaCl [88]
***Sarcocornia perennis***	Amaranthaceae	Dicot	Obligate halophyte	1.3 M NaCl [122]
***Schrenkiella parvula***	Brassicaceae	Dicot	Facultative extreme halophyte	0–400 mM NaCl [35]
***Suaeda aralocaspica***	Amaranthaceae	Dicot	Obligate halophyte	300 mM NaCl [37]
***Suaeda liaotungensis***	Amaranthaceae	Dicot	Obligate halophyte	0–1400 mM NaCl [40]
***Suaeda physophora***	Amaranthaceae	Dicot	Obligate halophyte	−1·34, −2·24, −3·13 MPa [25]
***Suaeda salsa***	Amaranthaceae	Dicot	Obligate halophyte	500 mM, 1500 mM NaCl [49]
***Tamarix spp.***	Tamaricaceae	Dicot	Facultative halophyte	1 M NaCl [122]
***Urochondra setulosa***	Poaceae	Monocot	Obligate halophyte	0–200 mM NaCl [123]
***Zygophyllum simplex***	Zygophyllaceae	Dicot	Facultative halophyte	0–100 mM NaCl [123]

### 4.2. Germination Recovery and Seed Priming: Convergent Pre-Germinative Checkpoints

The recovery of germination in halophyte seeds is a key determinant of their distribution and population persistence. Germination inhibition in halophytes depends on several factors, including the type and concentration, as well as the duration of exposure to saline conditions. Most recovery studies have used NaCl; however, different salt compositions, such as Na_2_SO_4_, may impose distinct osmotic and ionic stresses, leading to differential recovery responses [121].

Germination is restored when the external salinity level declines [35]. Reversible inhibition has been observed over a range of salt concentrations, from moderate salinity levels of 150–300 mM NaCl to high concentrations of 300 mM NaCl, and in some Amaranthaceae species, even up to 1 M NaCl [22,122] (Table 2). Short-term exposure, from hours to several days, generally allows high recovery percentages, whereas prolonged exposure may reduce recovery due to ionic toxicity and/or damage to the cell membranes.

The recovery of germination after exposure to NaCl has been reported in several species [88]. For example, *S. parvula* does not germinate at or above 200 mM NaCl but initiates germination after the salt concentration is reduced, indicating that it enters a quiescent rather than a lethal state. Several Amaranthaceae species (Table 2) recover even after exposure to excessive NaCl concentrations, such as 1000 mM NaCl, although recovery declines when exposure is prolonged [2]. The ability of halophyte seeds to enter quiescence under high salinity conditions in order to preserve viability is a survival strategy shaped by evolution. However, this adaptive capacity may vary depending on the environmental legacy inherited from the parent plant. At similar NaCl concentrations, seeds originating from halophytic habitats germinate at significantly higher levels than those from non-saline regions [18,124].

These findings indicate that germination recovery depends not only on salinity level but also on the salt source (i.e., the salt ions contributing to salinity), salt concentration, and duration of exposure. This dynamic response enables halophytic seeds to tolerate transient saline conditions and germinate when environmental conditions become favorable.

Seed priming is a promising biotechnological method for overcoming salinity during seed germination. It involves controlled pre-germinative activation, in which metabolic and hormonal processes are initiated without irreversible radicle protrusion. It is a simple, inexpensive, and effective pre-sowing technique that enables plants to better tolerate various abiotic stresses that limit plant establishment [125]. In this technique, dry seeds are treated with water for a certain period and then dried until they are sown again. This process activates the germination stage prior to radicle formation [126]. It has long been used by farmers, because primed seeds often show enhance germination [127]. Priming is not limited to hydropriming. Other methods, such as halopriming, hormonal priming, nanopriming, nutripriming, and osmopriming with mannitol, have also been used. These methods help researchers study seed germination under stressful conditions [128,129,130,131]. Some studies have reported that melatonin-primed halophyte seeds germinate better under salt conditions [71,123,132]. For example, melatonin priming of obligate halophyte *Suaeda corniculata* seeds increased antioxidant enzyme activity and promoted reserve mobilization during germination under salinity stress [38]. In addition, halopriming of seeds from two *Arthrocnemum* species improved germination under high salinity (e.g., 600 mM NaCl) [14]. Interestingly, moderate long-term salt exposure can act as a natural form of priming.

Recent developments have demonstrated the potential of physical priming techniques. Plasma technology is efficient, low-cost, fast, and environmentally friendly, and it can improve seed quality, promote growth, increase yield, and enhance tolerance to abiotic stress [133]. Germination percentages of habitat-indifferent halophyte *Prosopis koelziana* were reduced in 100 mM and 200 mM NaCl. However, these percentages improved by more than 2.5 times after 8 min of plasma treatment [32].

Seed priming and germination recovery share a common physiological basis before radicle emergence under favorable conditions. In both cases, seeds undergo partial metabolic activation, hormonal balance, and cellular repair. Thus, seed priming reflects the physiological logic underlying germination recovery. It enables seeds to initiate metabolic readiness while postponing radicle protrusion until environmental constraints, such as salinity, are alleviated. Although seed priming is an artificial intervention and germination recovery is a natural ecological process, both share conserved pre-germinative mechanisms. In both situations, seeds remain ready for development but delay radicle growth until conditions improve. This connection provides a framework for understanding how halophyte seeds respond to environmental signals to control germination timing. It also highlights the potential applications of ecological insights from halophytes for improving agricultural seed technologies. Therefore, germination recovery in halophytes can be considered an evolutionarily optimized form of preparation that delays irreversible growth until environmental constraints are removed.

## 5. Utilization of Halophyte Seeds in a Wide Range of Industrial Applications

The biochemical diversity of halophyte seeds and their capacity to develop in saline soil confer significant industrial relevance [134]. Several halophyte seeds can be used for biodiesel production, supporting sustainable energy production [33]. For example, *Salicornia* species have potential as liquid biofuel feedstocks [135,136,137,138].

Halophyte seeds play a key role in climate change by improving the fertility of arid regions. The elemental compositions of obligate halophytes *Anthrocnemum macrostachyum* and *A. meridionale* seeds, including K, Ca, Fe, Cu, Zn, Na, and Mg, has been reported to exceed that of *Chenopodium quinoa* while maintaining low concentrations of heavy metals [14]. These characteristics support their potential as nutrient-rich food sources. Additionally, extracts from halophyte seeds have been reported to be used for the synthesis of natural-based medicinal components for cancer treatment [139]. For instance, seed extracts of *Lasiurus scindicus* and *Panicum turgidum* function as biogenic agents for the synthesis of silver nanoparticles (AgNPs), which exhibit bioactivity against cancer cells and bacteria [27]. The seed mucilage from several halophyte species possesses strong adhesive properties, indicating potential use as a biodegradable glue [54].

Although molecular studies of halophyte seeds remain limited, recent genomic advances, including the sequencing of *S. parvula* and *E. salsugineum*, have facilitated research on stress tolerance mechanisms [116,140,141]. Furthermore, gene-editing technologies, such as CRISPR/Cas9, provide new opportunities to investigate seed biology. This technology transfers stress-resilient features into crops to improve salt tolerance [57].

These biofuel and medical applications are valuable for ecological restoration. Using halophyte seeds to rehabilitate salt-affected ecosystems and degraded soils, Shamsutdinov and Shamsutdinov (2008) emphasized that integrating halophyte germination strategies into restoration programs could increase biodiversity and productivity in degraded areas, particularly in the saline regions of Central Asia and Russia [142]. This information demonstrates the importance of understanding the germination recovery of halophytes. This is not only important for plant biology but also critical for global food security, sustainable land management, and ecosystem resilience to climate change.

## 6. Conclusions

This review highlights that, among halophyte groups, seed responses to salinity include not only tolerance but also regulatory strategies underlying active germination control. Halophyte seeds have structural, physiological, and molecular adaptations that help them survive and germinate in high-salinity environments [84]. These adaptations include specialized seed coats and mucilage production, which regulate water uptake and ion diffusion. Seed heteromorphism diversifies germination timing, whereas dormancy-like mechanisms prevent premature germination under salt stress. At the physiological level, halophytes maintain a dynamic balance between ABA and GA that regulates seed germination. ROS signaling further contributes to stress tolerance and the maintenance of genomic integrity. Salt-induced quiescence and rapid resumption of germination after stress alleviation are crucial adaptive responses. Together, these strategies enable halophyte seeds to survive in highly saline habitats. Halophytes are therefore valuable resources for expanding agriculture into saline and degraded lands.

## Data Availability

Data sharing is not applicable to this article as no new datasets were created or analyzed.
